# Intra-abdominal fat accumulation is an important predictor of metabolic syndrome in young adults

**DOI:** 10.1097/MD.0000000000022202

**Published:** 2020-09-11

**Authors:** Masakazu Kobayashi, Sayaka Ogawa, Jun Tayama, Ikuko Sagara, Atsushi Takeoka, Peter Bernick, Tetsuya Kawano, Norio Abiru, Masaki Hayashida, Susumu Shirabe

**Affiliations:** a Center for Health and Community Medicine, Nagasaki University, Nagasaki; b Faculty of Human Science, Waseda University, Saitama; c Student Accessibility Office, Nagasaki University; d Department of Endocrinology and Metabolism, Nagasaki University Graduate School of Biomedical Sciences; e Akiyama Hospital, Nagasaki Japan; f Research Center for the Control and Prevention of Infectious Diseases, Nagasaki University, Nagasaki, Japan.

**Keywords:** bio-impedance, intra-abdominal fat, metabolic syndrome, visceral, young

## Abstract

Metabolic syndrome (MetS), mainly caused by intra-abdominal fat (IAF) accumulation, is an important risk factor for cardiovascular disease. The prevalence of MetS increases rapidly after the age of 40 years, and it is presumed that there is a substantial proportion of MetS in younger age groups. However, the association of IAF with MetS in adults aged 20 to 30 years has not been fully investigated.

This study aimed to determine the prevalence of MetS and to verify whether IAF accumulation is associated with other MetS-related metabolic disorders including dyslipidemia, high blood pressure, and high blood glucose among the Japanese population in their 20s.

In this cross-sectional study, IAF area (IAFA) and MetS-related metabolic parameters were evaluated in university students in their 20s (n = 1822, 21.5 ± 1.5 years). IAFA was measured using a non-invasive device, DUALSCAN, which can be readily measured through the dual impedance method. The participants were divided into four groups according to IAFA: 0–49.9, 50–74.9, 75–99.9, and ≥100 cm^2^.

MetS was prevalent in 3.3% and 0.0% of the males and females, respectively, according to the Japanese criteria of MetS. The sex- and lifestyle-adjusted odds ratios (ORs) for the three metabolic component levels of Mets were elevated in the larger IAFA groups compared to the smallest IAFA group, according to the level of IAFA. The levels particularly increased in participants with abdominal obesity, defined by both, IAFA and waist circumference rather than by waist circumference alone.

IAF accumulation was significantly associated with MetS-related metabolic disorders in young adults. An evaluation of IAFA may contribute to the early prediction of the risk of developing MetS in the future.

## 1 Introduction

Metabolic syndrome (MetS), mainly caused by intra-abdominal fat (IAF) accumulation, is an important risk factor of cardiovascular disease; the incidence is increasing annually.^[1–3]^ In Japan, the clinical criteria for MetS required the presence of IAF accumulation (waist circumference [WC] of ≥ 85 cm and ≥ 90 cm for males and females, respectively, which corresponds to IAF accumulation of ≥ 100 cm^2^), and any 2 of the following three mild metabolic disorders:

(1) dyslipidemia,
(2) increased blood pressure (BP), and
(3) elevated fasting plasma glucose (FPG).^[4,5]^

At the WC cutoff point, the WHO and European criteria consider both, IAF accumulation and subcutaneous fat accumulation, while the Japanese criteria emphasize on IAF accumulation. The WC cutoff points are reversed in males and females in Japan, because the amount of subcutaneous fat is greater in females than in males with the same IAF; however, there is substantial individual variation.^[5]^ While these criteria are well defined, the cut off points of some of the variables pertaining to the diagnostic items, such as WC, FPG level, and high-density lipoprotein-cholesterol (HDL-C) level are still controversial.^[6]^ A Japanese study conducted between 2015 and 2017 reported that obesity was prevalent in 30.7% or 21.9% of males and females, respectively, and MetS was prevalent in 29.0% and 10.6% in the general adult population. Since the prevalence of MetS increases rapidly after the age of 40 years, it is presumed that there is a substantial proportion of individuals aged 20 to 30 years with pre-MetS, who are at high-risk of developing MetS. Although IAF accumulation is essential for the diagnosis of MetS in Japan, IAF has not been fully analyzed, particularly in young adults.

Abdominal computed tomography (CT) is the standard modality for evaluating IAF area (IAFA), but it has limitations due to radiation exposure problems, high cost, and the complexity of tracing the visceral fat region with the dedicated software. DUALSCAN is a non-invasive device, that can easily be used to measure the visceral fat through dual impedance analysis (dual bioelectrical impedance analysis [BIA]), and it has been reported to have good correlation with CT-measured IAFA.^[7]^

This study aimed to determine the prevalence of MetS among the Japanese population in their 20s and to verify whether IAF accumulation increases the risk of MetS in the future.

## 2 Methods

### 2.1 Study design and participants

This cross-sectional study was conducted between 2013 and 2018 and included 10691 participants in their 20s (6240 males and 4451 females). All the participants were students at Nagasaki University. They underwent annual health checkups including anthropometric measurements, laboratory analyses, and IAFA measurement, using the dual BIA instrument, DUALSCAN (Omron Dual scan HDS-2000; Omron, Kyoto, Japan). Informed consent was obtained from 9155 participants for undergoing IAFA measurement using the dual BIA instrument. Fasting blood samples were obtained from 2037 participants. Finally, we analyzed 1822 participants in their 20s (1163 males and 659 females), who provided informed consent and had complete data.

### 2.2 Definition of metabolic syndrome

In this study, we used the criteria proposed by the Japanese Committee for the Diagnostic Criteria of Metabolic Syndrome in 2005.^[4,5]^ The clinical criteria for MetS requires the presence of IAF accumulation (WC of ≥ 85 cm and ≥ 90 cm in males and females, respectively, which corresponds to IAF accumulation of ≥ 100 cm^2^) and any 2 of the following three mild metabolic disorders:

(1) dyslipidemia (triglyceride [TG] ≥ 150 mg/dL and/or HDL-C < 40 mg/dL),
(2) increased BP (systolic blood pressure [SBP] ≥ 130 mmHg and/or diastolic blood pressure [DBP] ≥ 85 mmHg), and
(3) elevated blood glucose (FPG ≥ 110 mg/dL).

### 2.3 Anthropometric measurement and laboratory analyses

Anthropometric factors including height, body weight, WC, and BP were measured. Each participant was asked to wear light clothing and no shoes during the measurements. Height and body weight were measured to the nearest 0.1 cm and 0.1 kg, respectively, using an electronic scale with an attached stadiometer (TANITA DC-250). WC was measured at the midpoint between the lower costal margin and the level of the anterior superior iliac crest using a non-elastic tape (Mutoh W12A-88-15 J1S1). Abdominal obesity was defined as WC ≥85 cm for males and ≥90 cm for females or an IAF of ≥100 cm^2^. BP was measured serially with an electrical sphygmomanometer (Japan Precision Instruments Inc. DM-3000). Participants were asked to rest quietly for over five minutes prior to the BPmeasurement. The representative values were calculated as follows: two measurements were taken with the participant in a sitting position, and the lower SBP measurement was chosen as the representative value. Blood was collected from the antecubital vein after overnight fasting. FPG, TG, and HDL-C were analyzed using standard laboratory methods.

### 2.4 Dual BIA method and instrumentation

The dual BIA instrument calculates the cross-sectional area of IAF at the level of the umbilicus based on the measurement of the electrical potentials resulting from the application of small electrical currents in the two body compartments. The principle of IAFA determination using the dual BIA instrument has been described previously.^[8,9]^ Other details of the study method have also been described previously.^[8–12]^

### 2.5 Lifestyle assessment

We assessed the participants’ smoking status, drinking status, physical activity, and eating behavior using a self-reported questionnaire. Current smokers were defined as those smoking more than one cigarette per day, habitually. Current drinkers were defined as those drinking more than one alcohol drink per week, habitually. Physical activity was determined by asking the participants whether they belong to a sports clubs and/or if they walk more than 30 minutes per day. Eating behavior was evaluated based on the eating of breakfast almost every day.

### 2.6 Statistical analysis

All clinical data were summarized as means and standard deviations. An unpaired *t* test or chi-square test was used for comparison between groups. To evaluate the relationship between IAFA and the three diagnostic MetS criteria, the participants were divided into four groups according to the IAFA (A1: 0–49.9; A2: 50–74.9; A3: 75–99.9; and A4: 100 cm^2^) and IAFA/WC [IAFA (cm^2^)/WC (male, female) (cm): G1:-99/-84 or -89; G2:-99/85- or 90-; G3: 100-/-84 or -89; and G4: 100-/85- or 90-]. The crude odds ratios (ORs) and 95% confidence intervals (95% CIs) of any 2 of the 3 MetS diagnostic criteria, as a dependent variable, were then compared with the smallest IAFA (A1) and IAFA/WC-group (G1), as reference. Multivariate logistic regression was then conducted by sex, smoking status, drinking status, physical activity, and eating behavior using scores of the self-reported questionnaire as the adjusted variables. The significance threshold was *P* < .05. All analyses were performed using SPSS software, version 23.0 (SPSS Inc., IL).

### 2.7 Ethical issues

This study was approved by the research ethics committee of the Nagasaki University (approval number: 15013069), and informed consent was obtained from all participants.

## 3 Results

### 3.1 Participant characteristics

Table 1 shows the characteristics of the study population, with the prevalence of MetS and metabolic parameters. MetS was prevalent in 3.3% and 0.0% of the males and females, respectively, according to the Japanese MetS criteria. The IAFA was 47.0 ± 25.3 cm^2^ and 33.1 ± 15.0 cm^2^ for the males and females, respectively. Age, BW, BMI, MetS prevalence, WC, IAFA, SBP, DBP, TG, and FPG were significantly higher while HDL-C was significantly lower in males.

**Table 1:**
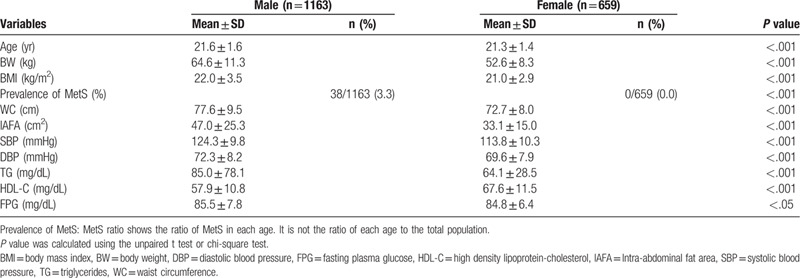
Prevalence of metabolic syndrome and metabolic parameters according to the Japanese criteria.

### 3.2 Comparison between participants with and without abdominal obesity

Comparison between participants with abdominal obesity defined according to WC ≥ 85 cm and IAFA ≥ 100 cm^2^ showed that MetS was prevalent in 18.7% or 0.0% of 203 males or 20 females with abdominal obesity (defined based on WC) and 41.0% or 0.0% of 39 males or 4 females (defined by IAFA), respectively (Tables 2 and 3). The values of BW, BMI, WC, IAFA, SBP, and DBP in males and those of BMI, WC, IAFA, and TG in females were higher in participants with abdominal obesity defined by IAFA than in those defined by WC (Tables 2 and 3).

**Table 2:**
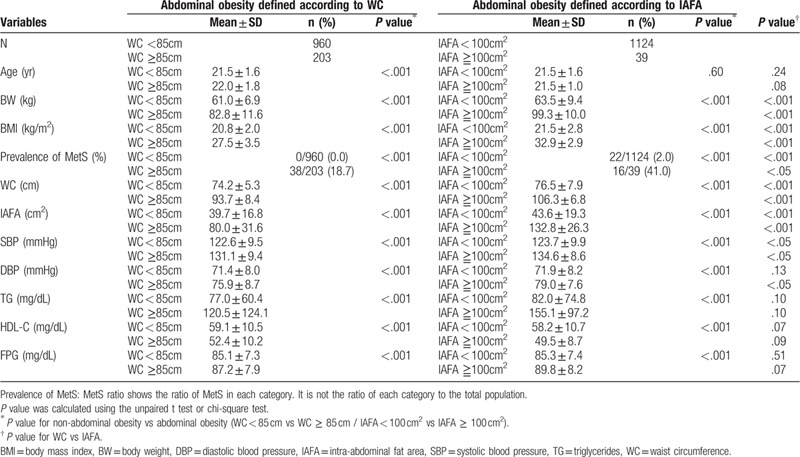
Comparison between males with and without abdominal obesity.

**Table 3:**
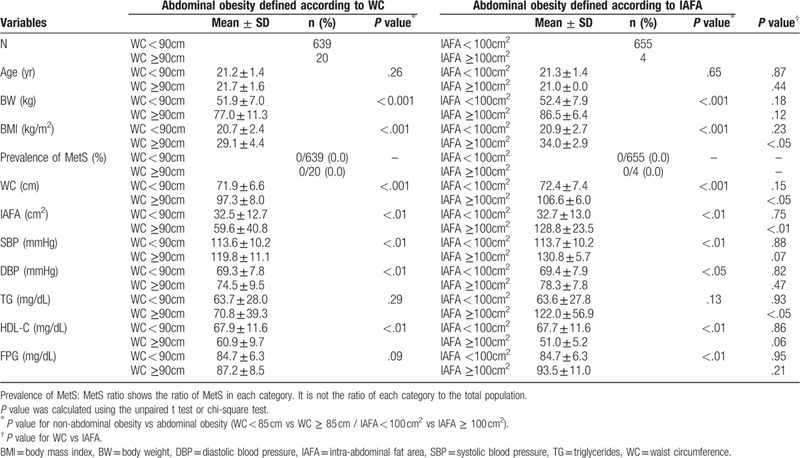
Comparison between females with and without abdominal obesity.

We further compared the levels of MetS-related metabolic parameters between participants with and without abdominal obesity. There were significant differences in all parameters in those with abdominal obesity defined by IAFA except for age in males, and age and TG in females (Tables 2 and 3).

### 3.3 Relationship between IAFA subgroups and MetS-related disorders

To evaluate the relationship between the levels of IAFA and MetS-related metabolic disorders including dyslipidemia, high BP, and high blood glucose, the participants were divided into 4 subgroups according to their IAFA designated groups, 1 through 4 (A1–4). Compared with group 1 (the smallest IAFA-group [A1]) values, the crude ORs and 95% CIs of MetS-related metabolic disorders were higher in all the groups with higher levels of IAFA (Table 4). After adjusting for sex, current smoking, current drinking, physical activity, and eating behavior, the significance of ORs were preserved (A2: OR: 4.80, 95% confidence interval [CI]: 2.42–9.51;A3: OR: 7.34, 95% CI: 3.01–17.89; A4: OR: 37.56, 95% CI: 16.06–87.80).

**Table 4:**
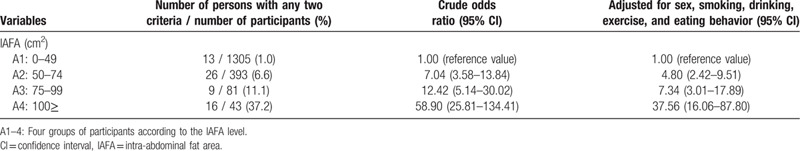
Relationship between intra-abdominal fat area and the risk of any two of the three diagnostic criteria for metabolic syndrome.

### 3.4 IAFA or WC as an index of visceral fat accumulation

To evaluate the usefulness of the IAFA and WC as an index of abdominal obesity, the participants were divided into four groups according to 2 distinct definitions of abdominal obesity in the IAFA and WC designated groups, 1 through 4 (G1: IAFA < 100 cm^2^ and WC < 85 cm [male]/ < 90 cm [female]; G2: IAFA < 100 cm^2^ and WC ≥ 85 cm [male]/≥ 90 cm [female]; G3: IAFA ≥ 100 cm^2^ and WC < 85 cm [male]/ < 90 cm [female]; G4: IAFA ≥ 100 cm^2^ and WC ≥ 85 cm [male]/≥ 90 cm [female]). There were no participants in the G3 group in the current study. Compared with the G1 (no abdominal obesity by both, IAFA and WC) values, the crude ORs and 95% CIs were elevated in G2 and G4, and were markedly increased in G4 (defined by both, IAFA and WC) rather than in G2 (WC alone) (G2: OR: 8.42, 95% CI: 4.76–15.21; G4: OR: 35.85, 95% CI: 17.28–74.37). After adjusting for sex, current smoking, current drinking, physical activity, and eating behavior, the significance of ORs was preserved in G2 and G4 (G2: OR: 5.66, 95% CI: 3.11–10.30; G4: OR: 24.56, 95% CI: 11.56–52.19) (Table 5).

**Table 5:**
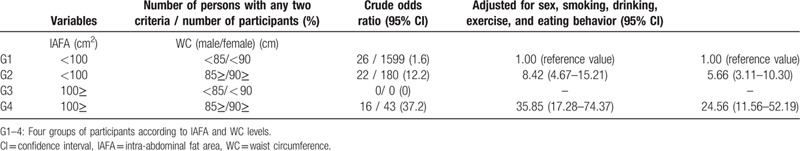
Relationship between intra-abdominal fat area /waist circumference and risk of any 2 of the 3 MetS diagnostic criteria.

### 3.5 Discussion and Conclusions

The present study revealed that MetS was prevalent in 3.3% and 0.0% of the males and females, respectively, in Japanese adults aged 20 to 30 years; the risk of MetS related disorders increased in young adult who had abdominal obesity.

Our results have shown that a certain population of young adult males, but not females has MetS in Japan. Few studies had investigated the risk of MetS in young adults. In these previous reports, the prevalence of MetS ranged from 3.7% to 10%.^[3,13]^ Another study reported an increasing IAF distribution in boys, but not girls in Japanese non-obese children aged older than 10 years.^[14]^ We have previously reported the relationship between IAF accumulation and hypertension in males, but not in females in our university students.^[12]^ Although there could be a sex difference in IAFA in Japanese young adults, our study revealed that almost all levels of MetS components were significantly elevated in both, males and females with abdominal obesity. These findings indicate that a substantial proportion of those aged 20 to 30 years with pre-MetS have a risk of developing MetS in the future, irrespective of sex.

In the clinical setting in Japan, IAF accumulation is often evaluated by measuring WC at the umbilical level.^[14]^ Several previous studies in Japan demonstrated a strong correlation between the level of umbilical WC and the risk of MetS^[15,16]^; however, this method is not used internationally.^[17–20]^ The Japan Obesity Society proposed a standard WC ≥ 85 cm for males and ≥ 90 cm for females as the main diagnostic criteria of MetS, corresponding to an IAF accumulation of ≥ 100 cm^2^ based on a study in adults aged approximately 55 years.^[21]^ In Japan, metabolic health check-up diagnosis is heavily based on the WC. However, recently, even if the WC is within the standard limit, the existence of “hidden metabolism” with dyslipidemia, high BP, and high blood sugar level has been regarded as a problem. Approximately more than 9 million Japanese aged over 40 years have “hidden metabolism”.^[22]^ The current method of taking WC measurements first overlook the “hidden metabolism” and cannot identify the risks for other diseases such as cardiovascular disease or stroke. Thus, urgent measures against “hidden metabolism” are needed in adults. In this context, it is questionable whether the WC levels in adults can be used to diagnose MetS in young adults in their 20s or to predict MetS-related metabolic disorders, which can consequently lead to the development of MetS in the future. To evaluate the usefulness of the definition of MetS by WC, corresponding to IAF accumulation in young adults, we compared several MetS-related metabolic parameters between participants with and without abdominal obesity, defined by WC and IAFA using a non-invasive device, DUALSCAN.

The mean level of IAF in the group with abdominal obesity defined by WC was only 80.0 ± 31.6 cm^2^, and was significantly smaller than that by IAFA (132.8 ± 26.3 cm^2^*P* < .01), indicating that the definition of abdominal obesity by WC was overestimated. The definition by IAFA ≥ 100 cm^2^ was more predictable for the diagnosis of MetS among participants with abdominal obesity compared to that by WC (18.7% (38/203) vs 41.0 (16/39), *P* < .05), since the number of those with abdominal obesity estimated by WC was significantly larger than that by IAFA. Further, almost all the values of MetS components were significantly elevated based on the levels of IAFA. Interestingly, in our study, the risk markedly increased in participants with abdominal obesity defined by both, IAFA and WC rather than by WC alone. Taken together with these results, the IAFA could be a better index of the definition of abdominal obesity rather than WC as one of the criteria of MetS in young adults in their 20s.

The present study has several strengths, including the direct assessment of IAF using DUALSCAN; this allowed for precise analysis of the relationship between IAF and MetS in adults in their 20s. In addition, we had a reasonable sample size (> 1000 subjects). Nevertheless, this study also had a limitation. The study subjects were chosen from one place (Nagasaki university), which was not representative of the general population. However, we confirm a similar mean BMI between our participants and the participants in the National Nutrition Survey in Japan (annual report of the National Health and Nutrition Survey in 2013–2017).^[23]^ This indicates that our results could be representative of the general population.

In conclusion, the MetS among the Japanese population in their 20s was less prevalent than those in adults. However, IAF accumulation was significantly associated with MetS-related metabolic disorders in young adulthood. Thus, IAF measurements in young adults may be useful for the identification of those at high-risk of developing MetS in later life.

## Acknowledgments

The authors thank Ms. Mayumi Maeda, Ms. Yoko Seike, Ms. Yuko Kuroki, and Ms. Kana Yoshida of the Center for Health and Community Medicine, Nagasaki University, Nagasaki, Japan for their technical assistance. We would like to thank Editage (www.editage.com) for English language editing.

## Author contributions

MK and SO designed and coordinated the study; JT, IS, TK, MH, and SS provided the information about the physiological variables; SO and JT analyzed the data; PB and NA interpreted the data. All authors have read and approved the final manuscript.
